# Discovery of rare cells from voluminous single cell expression data

**DOI:** 10.1038/s41467-018-07234-6

**Published:** 2018-11-09

**Authors:** Aashi Jindal, Prashant Gupta, Jayadeva, Debarka Sengupta

**Affiliations:** 1 Department of Electrical Engineering, Indian Institute of Technology Delhi, Hauz Khas, Delhi, 110016 India; 2 Center for Computational Biology, Indraprastha Institute of Information Technology, Delhi, 110020 India; 3 Department of Computer Science and Engineering, Indraprastha Institute of Information Technology, Delhi, 110020 India

## Abstract

Single cell messenger RNA sequencing (scRNA-seq) provides a window into transcriptional landscapes in complex tissues. The recent introduction of droplet based transcriptomics platforms has enabled the parallel screening of thousands of cells. Large-scale single cell transcriptomics is advantageous as it promises the discovery of a number of rare cell sub-populations. Existing algorithms to find rare cells scale unbearably slowly or terminate, as the sample size grows to the order of tens of thousands. We propose Finder of Rare Entities (FiRE), an algorithm that, in a matter of seconds, assigns a rareness score to every individual expression profile under study. We demonstrate how FiRE scores can help bioinformaticians focus the downstream analyses only on a fraction of expression profiles within ultra-large scRNA-seq data. When applied to a large scRNA-seq dataset of mouse brain cells, FiRE recovered a novel sub-type of the pars tuberalis lineage.

## Abstract (web summary)

Algorithms designed to find rare cells in single cell RNA-seq data sets cannot cope with data sets containing tens of thousands of cells. Here the authors present Finder of Rare Entities (FiRE), an algorithm that uses the Sketching technique to assign a rareness score to every expression profile in large RNA-seq data sets.

## Introduction

Unabated progress in technology over the past years has made transcriptome analysis of individual cells^1^ a reality. Cells, the basic units of life, and building blocks for complex tissues, are shaped by multiple factors that affect their identity. Given a heterogeneous cell population, single-cell RNA-sequencing (scRNA-seq) screens gene expression levels in individual cells, as opposed to measuring their population-level average expression-signature using, say, bulk RNA-sequencing.

Comprehensive characterization of all major and minor cell types in a complex tissue requires processing several thousand single cells^2^. In other words, larger sample sizes better the odds of capturing minor cell subpopulations in a tissue. It is primarily because a large number of cell type-specific transcripts are not detected in the sequencing, due to the failure at the amplification stage. As a result, a small number of cell type-specific genes often fail to influence the downstream analysis regime sufficiently. Quite fortunately, recent discovery of the droplet-based single-cell transcriptomics has enabled the parallel profiling of tens of thousands of single cells, at a significantly reduced per-cell cost. To date, many studies have been published with reported transcriptomes ranging between ~20 k and ~70 k in number^3–7^.

The advent of single-cell transcriptomics has made rare cell discovery a mainstream component in the downstream analysis pipeline. Rare cells represent minor cell types in an organism. When the number of profiled cells are in the hundreds, even an outlier cell (singleton) deserves attention. With the increase in throughput capabilities, however, the focus shifts to the discovery of minor cell types rather than mere singletons. Examples of rare cell types include circulating tumor cells, cancer stem cells, circulating endothelial cells, endothelial progenitor cells, antigen-specific T cells, invariant natural killer T cells, etc. Despite low abundance, rare cell populations play an important role in determining the pathogenesis of cancer, mediating immune responses, angiogenesis in cancer and other diseases, etc. Antigen-specific T cells are crucial to the formation of immunological memory^8–10^. Endothelial progenitor cells, which originate from the bone marrow, have proven to be reliable biomarkers of tumor angiogenesis^11,12^. Stem cells have an ability to replace damaged cells, and to treat diseases like Parkinson’s, diabetes, heart diseases, etc.^13^. Circulating tumor cells offer unprecedented insights into the metastatic process with real-time leads for clinical management^14^.

Algorithms for detecting rare cell transcriptomes are scarce. Prominent among these are rare cell-type identification (RaceID)^15^ and GiniClust^16^. RaceID involves computationally expensive parametric modeling for the detection of outlier expression profiles. It uses unsupervised clustering as an intermediate step to define populous cell types, which in turn are used to determine outlier events (cells). GiniClust, on the other hand, uses a rather straightforward two-pronged algorithm. First, it selects informative genes using the Gini index. It then applies a density-based clustering method, density-based spatial clustering of applications with noise (DBSCAN)^17^, to discover outlier cells. Notably, both RaceID and GiniClust use clustering to distinguish between major and minor cell types. In fact, both these algorithms compute the distance between each pair of cells. A number of such design choices make both these algorithms slow and memory inefficient for oversized scRNA-seq data.

We propose Finder of Rare Entities (FiRE), a conspicuously fast algorithm to estimate the density around each subjected multidimensional data point. This is achieved by using the Sketching technique^18,19^ as the workhorse algorithm. Unlike the existing techniques, FiRE assigns a rareness score to each of the individual expression profiles, thus giving the user a choice for focusing only on a small set of potentially rare cells.

We evaluated FiRE on a number of real and simulated datasets. FiRE discovered some acutely rare cell types from a large scRNA-seq mouse brain dataset. We cross-referenced our findings with in situ hybridization data obtained from the Allen Mouse Brain Atlas^20^. We also demonstrated the efficacy of FiRE in delineating human blood dendritic cell sub-types using ~68 k single-cell expression profiles of human blood cells.

## Results

### Overview of FiRE

Both RaceID and GiniClust use clustering in some form, as an intermediate step for detecting rare cells. Clustering by its very nature is often dependent on a number of sensitive parameters and works inefficiently as density varies across data points. Another major problem is to decide the resolution of group identities. Often, multi-level clustering becomes essential as minor clusters get overlooked on the first pass^5^. This happens since other major cell types influence the expression variance in a data. We asked if it is possible to develop an original, monolithic algorithm which bypasses clustering, while straightforwardly estimating the rareness of a cell (multidimensional data point).

**Fig. 1:**
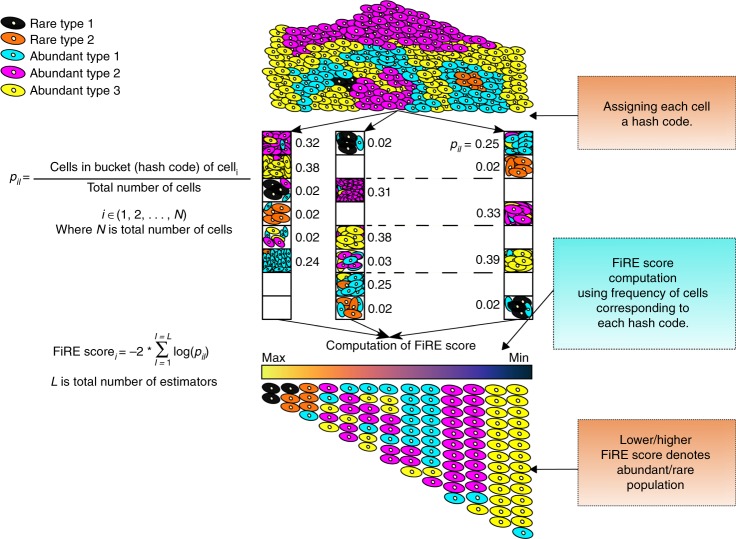
Overview of FiRE. The first step is to assign each cell to a hash code. A hash code can be considered as an imaginary bucket since multiple similar cells can share a hash code. For the robustness of rarity estimates, the hash code creation step is repeated for *L* times. For each cell *i* and estimator *l*, *p*_*il*_ is computed as the probability for any point to land in the bucket of *i*. The second step of the algorithm involves combining these probabilities to obtain a rareness estimate for each cell

To circumvent the above issues, we propose FiRE to identify rare cell types. Design of FiRE is inspired by the observation that rareness estimation of a particular data point is the flip side of measuring the density around it. The algorithm capitalizes on the Sketching technique^18,19^, a powerful technique for low-dimensional encoding of a large volume of data points (Methods). It works by randomly projecting points to low-dimensional bit signatures (hash code), such that the weighted L_1_ distance between each pair of points is approximately preserved. The computation involved in the creation of hash codes is linear with respect to (w.r.t.) the number of individual transcriptomes. A hash code can be imagined as a bucket that tends to contain data points which are close by in the concerned hyper-dimensional space. The cell originating from a large cluster shares its bucket with many other cells, whereas a rare cell shares its bucket with only a few. To this end, FiRE uses the populousness of a bucket as an indicator of the rareness of its resident data points. To ward off biases, FiRE uses several such rareness estimates to arrive at a consensus rareness score for each of the studied cells. This score is termed as the FiRE score. The Methods section contains an elaborate explanation of the various steps involved in FiRE (Supplementary Methods). Figure 1 depicts a visual interpretation of FiRE.

**Fig. 2:**
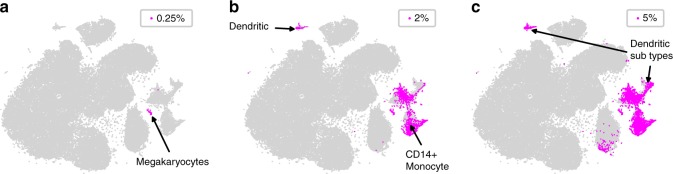
FiRE discovers cells with varying degrees of rareness. In the ~68 k PBMC data^3^, minor cell populations with different grades of rarity show up with an increase in the number of selected rare cells. **a**–**c** The top 0.25, 2 and 5% cells, respectively, selected on the basis of FiRE scores are highlighted

FiRE assigns a continuous score to each cell, such that outlier cells and cells originating from the minor cell populations are assigned higher values in comparison to cells representing major subpopulations. A continuous score gives users the freedom to decide the degree of the rareness of the cells, to be further investigated. To illustrate this, we applied FiRE on a scRNA-seq data containing ~68 k peripheral blood mononuclear cells (PBMCs), annotated based on similarity with purified, well-known immune cell sub-types^3^ (Methods). Authors of the study performed unsupervised clustering of the cells and annotated the clusters based on previously known markers (Supplementary Figure 1a). We overlaid FiRE scores on the two-dimensional (2D) map reported as part of the study (Supplementary Figure 1c). The top 0.25% highest FiRE scores exclusively corresponded to the smallest, unambiguously annotated cluster harboring megakaryocytes (Fig. 2a). Of note, megakaryocytes represent only 0.3% of the entire set of the profiled cells. As we increased the proportion from 0.25% to 2.0% and subsequently 5.0%, the next batches of minor cell sub-types made their way into the extended set of rare cells. These include sub-classes of monocytes and dendritic cell sub-types (Fig. 2b, c). The case study highlights the utility of FiRE in discovering cells with varying degrees of rareness.

While a continuous score is helpful, sometimes a binary annotation about cell rarity eases out the analysis. To this end, we introduced a thresholding scheme using the properties of the score distribution (Methods). Supplementary Figure 1b highlights cells in the ~68 k PBMC data which are detected as rare going by the threshold-based dichotomization. As expected, a majority of the detected rare cells originated from known minor cell types such as megakaryocytes, dendritic cells and monocytes.

It should be noted that unlike GiniClust and RaceID, FiRE refrains from using clustering as an intermediate step to pinpoint the rare cells. Clustering is done at a later phase for delineating minor cell types from the detected rare cells.

### FiRE recovers artificially planted rare cells

**Fig. 3:**
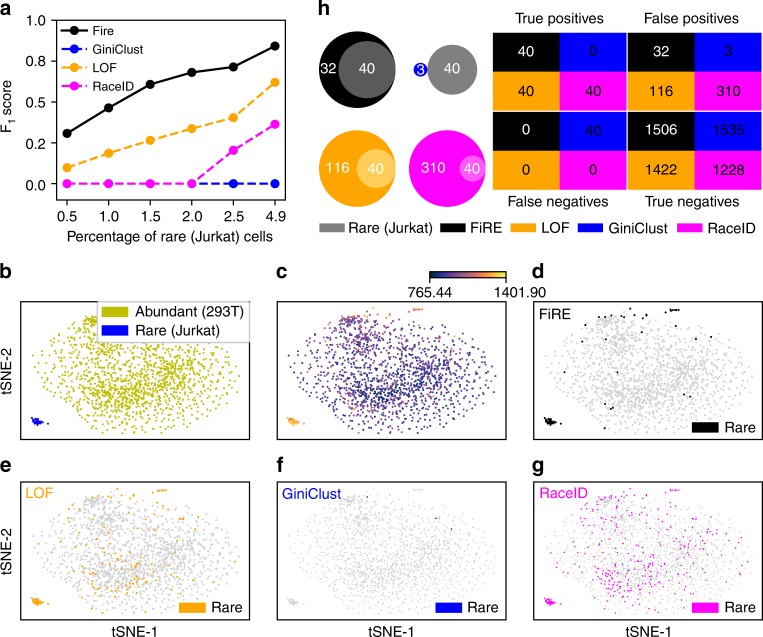
Detectability of minor cell types in a simulated dataset consisting of Jurkat and 293T cells. **a** F_1_ scores were calculated w.r.t. the rare (Jurkat) population, while bioinformatically varying the proportion of artificially planted rare cells. Notably, both FiRE and LOF^21^ apply a threshold to their continuous scores for zeroing on the rare cells. On the contrary, GiniClust^16^ and RaceID^15^ provide binary annotations for cell rarity. **b** The t-SNE-based 2D embedding of the cells with color-coded identities. **c** FiRE score intensities plotted on the t-SNE-based 2D map. **d**–**g** The rare cells detected by various algorithms are highlighted. **h** Congruence of methods with known annotations. Note that the results shown in (**b**–**h**) correspond to a rare cell concentration of 2.5%

We designed a simulation experiment to evaluate the performance of FiRE in the presence of ground truth information pertaining to the cell-type identity. For this, we used a scRNA-seq data comprising 293T and Jurkat cells mixed in vitro in equal proportion (Methods)^3^. The authors exploited the single-nucleotide variant (SNV) profile of each cell to determine its lineage. We considered this genotype-based annotation scheme to be near confirmatory. With this data, we mimicked the rare cell phenomenon by bioinformatically diluting Jurkat cell proportion in the data. We varied the proportion of Jurkat cells between 0.5 and 5%. Besides GiniClust and RaceID, we compare FiRE with a rare event detection algorithm called local outlier factor (LOF). LOF is a widely used algorithm in the field of data mining. The performance of various methods was measured using F_1_ score (Methods) with respect to the minor population of the Jurkat cells. F_1_ score reflects the balance between precision and sensitivity. FiRE clearly outperformed LOF^21^, RaceID^15^ and GiniClust^16^ on each of the test cases (Fig. 3a). Notably, RaceID and GiniClust report dichotomized predictions for rare cells, whereas FiRE and LOF offer both continuous scores and binary prediction. FiRE implements an interquartile range (IQR)-based thresholding technique for the dichotomization (Methods).

We took a closer look at working of the methods at a rare cell concentration of 2.5%. We found FiRE scores of the rare cells to be unambiguously higher compared to the abundant cell type (Fig. 3c). Figure 3d–g marks the rare cells detected by each of the algorithms. Among all algorithms, FiRE displayed the highest level of congruence with the known annotations (Fig. 3h). Supplementary Figure 5 shows the congruence between each pair of methods.

To evaluate the performance of the techniques, we used two additional datasets: ~2.5 k embryonic stem cells (ESCs)^22^, and 288 mouse intestinal organoid cells data^15^ (Methods). FiRE and LOF could identify *Zscan-4* enriched, 2C-like cells as reported by the authors of the GiniClust algorithm^16^ (Supplementary Figure 4a). In addition, FiRE had the least overlap with RaceID which could not identify the 2C-like cell type. Supplementary Figure 4b depicts the performance of the various methods in detecting rare cell types in the secretory lineage of mouse small intestine, as reported by the authors of the RaceID algorithm. Both FiRE and LOF could detect almost all of the designated rare cell types including the goblet, tuft, paneth and enteroendocrine cells. GiniClust could detect only a fraction of these cells.

### FiRE is sensitive to cell type identity

A simulation study was designed to analyze the robustness and sensitivity of FiRE score with respect to the number of differentially expressed genes. We first generated an artificial scRNA-seq data consisting of 500 cells and two cell types. The minor cell type represented about 5% of the total population (Methods). We kept aside the differentially expressed (DE) genes which we selected through a stringent criterion. For every iteration of the experiments, we replaced a fixed number of non-DE genes by the pre-identified DE genes. We varied the count of differentially expressed genes between 1 and 150 to track the sensitivity of FiRE in detecting the minor population.

**Fig. 4:**
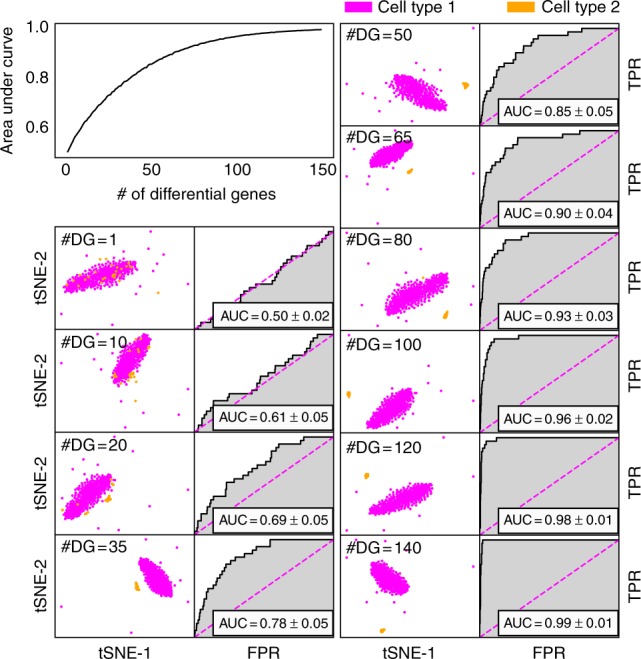
The sensitivity of FiRE to cell-type identity. On the scRNA-seq data simulated using R package splatter^33^, FiRE starts recognizing the minor cell type correctly, as soon as the number of differentially expressed genes becomes adequate to give rise to a tiny cluster representing the minor cell subpopulation. The top-left corner figure serves as a legend for the subsequent AUC-ROC plots. Each t-SNE and ROC plot pair serves as a representative of the 1000 repetitions of the experiment concerning a specific number of differentially expressed genes. The AUC-ROC analysis was performed using cell-type annotations, and FiRE scores were assigned to individual cells

With the given set of DE genes, FiRE scores were obtained and used for computing the area under the curve of receiver operating characteristics (AUC-ROC) with respect to the minor population. For every count of DE genes, the aforementioned process was repeated 1000 times to report an average AUC-ROC (Fig. 4).

With a small number of DE genes, FiRE struggled to detect the minor cell population. However, FiRE predictions improved sharply when 20 or more DE genes were introduced. It reflects the robustness of FiRE against noise. A plausible explanation for the same could be that a small number of differential genes fail to stand out in the presence of cell type-specific expression noise (biological plus technical).

### FiRE is scalable and fast

Both RaceID and GiniClust are slow and incur significant memory footprints. For both these methods, clustering takes *O*(*N*^2^) time, where *N* is the number of cells. RaceID additionally spends enormous time in fitting parametric distributions for each cell–gene combinations. On the other hand, LOF requires a large number of *k*-nearest neighbor queries to assign an outlierness score to every cell. FiRE, on the other hand, uses Sketching^18^, a randomized algorithm for converting expression profiles into bit strings while preserving the weighted L_1_ distance between data points (Methods). The main advantage of randomized algorithms is that they usually save a lot of computational time. FiRE generates a rareness estimation of *N* cells in linear, i.e., *O*(*N*) time.

**Fig. 5:**
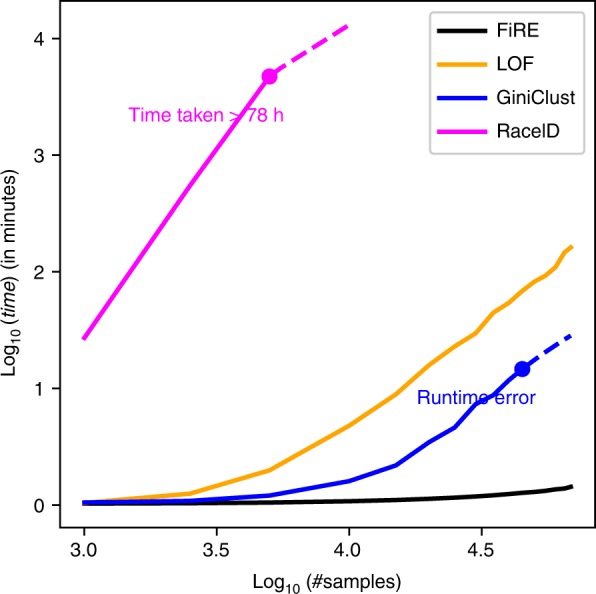
FiRE is fast. Execution time recorded for the four methods (FiRE, LOF^21^, RaceID^15^ and GiniClust^16^) while varying the number of cells from 1 k to ~68 k

We tracked the time taken by LOF, RaceID, GiniClust and FiRE, while varying the input data size (Fig. 5, and Supplementary Table 1), on a single core of a machine with a clock speed of 1.9 GHz, and 1024 GB DDR4-1866/2133 ECC RAM. FiRE turned out to be remarkably faster as compared to LOF, RaceID and GiniClust. For FiRE, we recorded ~26 s on the ~68 k PBMC data. GiniClust reported a runtime error, when the input expression profiles increased beyond ~45 k, while RaceID took ~79 h for just 5 k cells.

### FiRE enables discovery of new rare cell types in mouse brain

Often, clustering on the first pass fails to discover the minor subpopulations. The second pass of clustering, thus, becomes necessary on the first level clusters^5,23^. In fact, there is no clear directive about the number of levels required for the comprehensive charting of the cell types from a given scRNA-seq dataset. Clustering, being an NP-hard problem, requires significant computing power. Making it scalable for large data demands various compromises^24^. FiRE could be particularly helpful in addressing this challenge. To illustrate this, we used an existing scRNA-seq data containing ~20 k cells profiled from in and around the Arcuate–Median Eminence (Arc-ME) region of the mouse brain^5^.

**Fig. 6:**
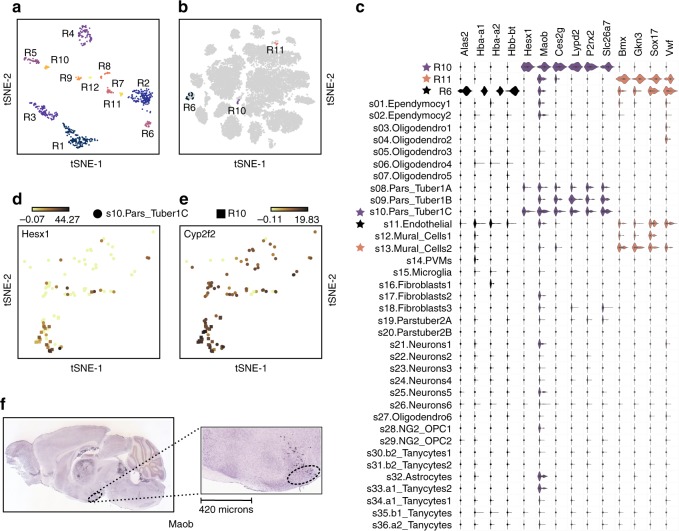
Identification of novel, rare cell types from mouse brain cell data^5^. **a** Clustering of rare cells detected by FiRE from the ~20 k mouse brain data using dropClust^24^. Predicted clusters numbered R1–R12 are shown in a t-SNE-based 2D map. **b** Three clusters (R6, R10, R11) out of the 12 clusters, characterized further, are marked on the two-dimensional t-SNE plot adopted from the original study^5^. Notably, all three cell subpopulations appear to be inscribed within some high-level clusters reported by Campbell et al.^5^. **c** Violin plots showing the expression distribution of some of the cell type-specific genes uniquely marking the newly discovered minor subpopulations. Minor cell subpopulations and their corresponding parent clusters have been prefixed with asterisks of the same color. **d** Cells in R10 express high levels of *Hesx1*. **e** R10 expressed *Cyp2f2*, a known marker of its parent sub-type s10-pars tuber 1c^5^. **f** *Maob* in situ hybridization in a sagittal view of Arc region of brain (scale = 420 µm), obtained from the Allen Mouse Brain Atlas^20^

DropClust-based clustering of the FiRE detected 727 rare cells (using IQR-based thresholding criterion) yielding 12 cell subpopulations (Fig. 6a). We labeled these cell groups as R1–R12. Among these, R6 (0.15%), R10 (0.12%) and R11 (0.057%) were found to be inscribed almost exclusively within some of the single, minor subpopulations reported by the authors of the original study (Supplementary Table 2). Cell counts in these clusters varied between 12 and 32. The cell-type identity of the newly observed clusters was discovered trivially by referring to their respective parent clusters as reported by Campbell et al.^5^ (Fig. 6b, Supplementary Figure 6). Clusters R10 and R11 originated from one of the sub-types of the pars tuberalis cells and mural cells, respectively. R6 emerged from the cluster containing endothelial cells.

We performed differential expression analysis to find cell type-specific genes for the newly retrieved cell subpopulations (Methods). Every cluster was found to have a number of differentially up-regulated genes, clearly distinguishing it from the remaining cell types (Fig. 6c). Cells in the R6 cluster displayed numerous erythrocyte markers including *Alas2*^25^ and various hemoglobin subunits (*Hba-a1*, *Hba-a2* and *Hbb-bt*). These cells co-clustered with the endothelial cells due to their hematopoietic origin. Cluster R10, which emerged as a sub-type of pars tuber type 1C, expressed *Cyp2f2*, a marker of its parent cell type (Fig. 6e). R10 did not get resolved even after applying a second level clustering, which otherwise yielded three sub-types including pars tuber 1C^5^. Also, we found R10 to be visually undetachable from its parent cluster. Cells from R10 displayed high expression levels of *Hesx1*, which is known to play an essential role in the formation of the pituitary gland^26^ (Fig. 6d). We also found a mural cell sub-type, i.e., R11, with a spectrum of cell type-specific markers. However, we could not zero in on its biological relevance. Upon cross-referencing with the in situ hybridization (ISH) data from the Allen Mouse Brain Atlas^20^, we could spot the presence of *Maob*, an R10 marker, in the arcuate hypothalamus (Arc) region (Fig. 6f).

We asked if any of the newly detected cell types harbor doublets. None of the cells from the three newly discovered clusters matched with the top thousand putative doublets as identified by DoubletFinder^27^ (Methods). Of note, only 31 out of the 727 FiRE predicted rare cells were enlisted among the thousand.

### FiRE resolves heterogeneity among dendritic cells

Dendritic cells (DCs) play a central role in antigen surveillance. DCs are among the rarest immune cell types, constituting about 0.5% of the PBMCs^28^. A recent study by Villani^29^ delineated six different sub-types of dendritic cells by analyzing the expression profiles of fluorescence-activated cell sorting (FACS) sorted population of DCs and monocytes. DC sub-types reported by the authors are as follows: CD141^+^ DCs (DC1), CD1C^+^_A conventional DCs (DC2), CD1C^+^_B conventional DCs (DC3), CD1C^−^CD141^−^ (DC4), DC5 and plasmacytoid DCs (DC6, pDCs)^29^.

**Fig. 7:**
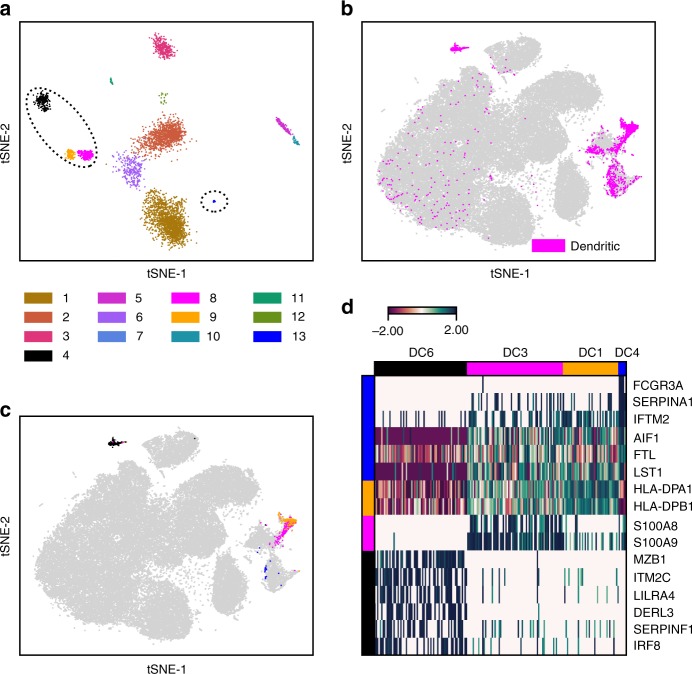
Human blood dendritic cell heterogeneity delineated by FiRE. **a** t-SNE-based 2D plot of rare cells detected by FiRE. Cells are color coded based on their cluster identity as determined by dropClust^24^. **b** Dendritic cells, annotated by the authors, are highlighted in the 2D map adopted from Zheng et al.^3^. **c** Dendritic cell sub-types (DC6, DC3, DC1 and DC4) detected from FiRE-reported rare cells (R4, R8, R9 and R13) are color coded as per (**a**). **d** Characterization of dendritic cell sub-types using markers reported by Villani^29^

We asked if some of these dendritic cell sub-types could be identified in unfractionated PBMC data. To this end, we applied FiRE on the ~68 k PBMC data. FiRE reported a total of 4238 rare cells (using IQR-based dichotomization), which we then clustered using dropClust^24^. Out of the 13 clearly distinguishable clusters (R1–R13), R4, R8, R9 and R13 exclusively consisted of dendritic cells as per the annotations provided by Zheng et al.^3^ (Fig. 7a, c, Supplementary Figure 2). For these 4 DC clusters, we conducted differential expression analysis to find the cell type-specific genes (Methods). By overlaying our differential genes with the ones reported by Villani^29^, we could confidently resolve four (DC1, DC3, DC4, DC6) out of the six sub-types reported by Villani ^29^ (Fig. 7d).

To summarize, when applied to the ~68 k PBMC data, FiRE helped in delineating four distinct DC sub-types, of which DC1, DC3 and DC4 were unresolved by the unsupervised clustering used in the original study^3^. In fact, in the *t*-distributed stochastic neighbor embedding (t-SNE)-based 2D embedding, these cell types were visually co-clustered within themselves or the monocytes.

## Discussion

Of late, single-cell transcriptomics has considerably refined our understanding about the true nature of cellular phenotype. It has also accelerated the discovery of new cell types. Most of these new cell types are rare since it is quite improbable for an abundant cell type to remain unobserved for a very long time. A truly rare cell type can only be found by profiling several thousands of cells^2^. While technological advances over the past years have enabled us to perform ultra high-throughput single-cell experiments, scalable methods for rare cell detection are nearly non-existent. FiRE attempts to fill that gap, with a number of pragmatic design considerations. Most notable among these is its ability to avoid clustering as an intermediate step. A typical clustering technique is not only time consuming, but also incapable of comprehensively charting the minor cell types in a complex tissue on a single go^5^.

While RaceID^15^ and GiniClust^16^ offer binary predictions, FiRE gives a rareness score to every individual expression profile. We demonstrated how these scores might help the users focus their downstream analyses on a small fraction of the input scRNA-seq profiles. A score is particularly helpful since a number of complex techniques such as pseudo-temporal analysis^30^, shared nearest neighborhood-based topological clustering^31^ et cetera are applicable only on a few hundreds of cells.

FiRE makes multiple estimations of the proximity between a pair of cells, in low-dimensional spaces, as determined by the parameter *M*. The notion of similarity for LOF^21^, on the other hand, is confounded by the arbitrary scales of the input dimensions. As a result, even though LOF consistently outperforms RaceID and GiniClust, it struggles to match the performance of FiRE.

FiRE, in principle, does not discriminate between an outlier and cells representing minor cell types. For clustering FiRE detected rare cells, in all our analyses, we adhered to dropClust^24^ that does not administer any special treatment to outliers. As a result, outlier cells, if any, get submerged into the minor cell clusters. However, one may wish to use hierarchical or density-based clustering techniques to flag outlier cells.

Doublet detection may be a potential application of FiRE. However, doublet rate may vary between 1% and 30%^32^ which is a considerably wide range and does not necessarily comply with the notion of cell rareness.

FiRE took ~31 s to analyze a scRNA-seq dataset containing ~68 k expression profiles. Such unrivaled speed, combined with the ability to pinpoint the truly rare expression profiles, makes the algorithm future proof.

## Methods

### Description of datasets

For the various analyses, we used five publicly available scRNA-seq datasets. For a simulation experiment of artificially planted rare cells, we used 293T and Jurkat cell data containing a total of ~3200 cells, with an almost equal number of representative transcriptomes of each type. The cells were mixed in vitro at equal proportions. Authors of the study resolved the cell types bioinformatically exploiting their SNV profiles^3^.

Our second data contained ~20 k scRNA-seq profiles from around the Arc-ME region of the mouse brain^5^. The authors grouped the neuronal cells into 34 clusters, and non-neuronal cells into 30 clusters through a two-pass clustering approach. We found the neuronal cell classification to be exhaustive and therefore focused on the 30 non-neuronal clusters.

We used a large-scale scRNA-seq data containing expression profiles of ~68 k PBMCs, collected from a healthy donor^3^. Single-cell expression profiles of 11 purified subpopulations of PBMCs were used as a reference for cell-type annotation.

We applied FiRE on a publicly available ~2.5 k mouse ESC data^22^. Mouse embryonic cells were sequenced at different points after the removal of leukemia inhibitory factor. Similar to Jiang et al.^16^, we used day 0 data where stem cells were undifferentiated. Data contained a total of 2509 cells.

Our fifth scRNA-seq data contained single-cell expression profiles of mouse intestinal organoids^15^. A set of 288 organoid cells were randomly selected and sequenced using a modified version of the cell expressions by linear amplification and sequencing (CEL-seq) method. Unique Molecular Identifiers (UMIs) were used to count transcripts.

### Data preprocessing

Mouse ESCs and mouse small intestine datasets were screened for low-quality cells. For mouse ESC data, cells having more than 1800 detected genes were selected for analysis. For the intestine dataset, the cutoff for the number of detected genes was set at 1200. The remaining datasets were already filtered.

For each dataset, genes which had a read count exceeding 2 in at least 3 cells were retained for downstream analysis. Each scRNA-seq data were normalized using median normalization. The 1000 most variable genes were selected, based on their relative dispersion (variance/mean) with respect to the expected dispersion across genes with similar average expression^3^. The normalized matrix was then log_2_ transformed after addition of 1 as a pseudo count.

The preprocessed mouse brain data was downloaded from the Single cell Portal (https://portals.broadinstitute.org/single_cell). We applied anti-log to all elements of the matrix before subjecting the same to dispersion-based gene selection.

### Simulation to assess sensitivity of FiRE to DE genes

To analyze the sensitivity of FiRE to cell-type identity, we generated an artificial scRNA-seq data using the splatter R package^33^. The following command was used to generate these data:

splatSimulate(group.prob = c(0.95, 0.05), method = ‘groups’, verbose = F, batchCells = 500, de.prob = c(0.4, 0.4), out.prob = 0, de.facLoc = 0.4, de.facScale = 0.8, nGenes = 5000).

The generated dataset had 500 cells and 5000 genes per cell. Out of the 500 cells, 472 cells represented the major cell type, whereas 28 cells defined the minor one.

Genes for which expression counts exceeded 2 in at least 3 cells were considered for analysis. The filtered data was log_2_ transformed after adding 1 as a pseudo count. On the transformed data, differential genes were detected using Wilcoxon’s rank sum test with the false discovery rate (FDR) cutoff of 0.05 and as an inter-group absolute value of fold-change cutoff of 2.32 (log_2_ (5)). The differentially expressed genes, which were 180 in number, were removed from the data and kept as a separate set. Genes with a *p* value of more than 0.05, 2387 in number, were kept as a separate set of non-differential genes.

### Sketches

A Sketch encodes a high-dimensional data point to a bit vector. The length of the bit vector is usually much smaller than the data dimension. These bit vectors are constructed using a randomized algorithm as outlined below: For each cell, *c* in a given scRNA-seq data, a Sketch or bit vector (containing 0 s and 1 s) of size *M* is generated by randomly selecting *M* genes at a time and applying thresholds on each of them. A threshold is a randomly chosen numeric value lying between the minimum and maximum values observed in a given expression matrix. A weight vector *w* is generated randomly such that $w \in {\Bbb R}^M$. The dot product between a Sketch and *w* is mapped to one of the predetermined hash codes using the modulo hashing technique. The hamming distance between a pair of Sketches approximates the L_1_ distance between their corresponding high-dimensional data points (cells)^18,19^.

### Steps involved in FiRE

FiRE is a two-stage algorithm. In the first stage, the Sketching process is repeated *L* times. On each pass, hash codes are generated for the entire set of expression profiles. A hash code can be thought as a bucket. Sketching ensures that cells which share their bucket are nearby in the original high-dimensional space. The density estimate for the *i*-th cell on the *l*-th pass is expressed as follows.

$$p_{il} = \frac{{{\mathrm{Number}}\,{\mathrm{of}}\,{\mathrm{cells}}\,{\mathrm{in}}\,{\mathrm{the}}\,{\mathrm{bucket}}\,\left( {{\mathrm{hash}}\,{\mathrm{code}}} \right)\,{\mathrm{containing}}\,{\mathrm{cell}}\,i}}{{{\mathrm{Total}}\,{\mathrm{number}}\,{\mathrm{of}}\,{\mathrm{cells}}}}.$$

Here *l* denotes the index of the estimator, where 1 ≤ l ≤ *L*. In other words, *p*_*il*_ is the probability that any randomly picked cell is assigned to the bucket that contains the *i*-th cell.

At the second stage, we aim at reducing the variance of our density estimates for the individual cells. This is important due to the intrinsic dimensionality of a typical scRNA-seq data. FiRE reduces the variance by combining the *L* density estimates for each cell. A FiRE score is defined as follows.

$${\mathrm{FiRE}}\,{\mathrm{score}}_i = - 2\mathop {\sum}\limits_{l = 1}^L {\mathrm{log}}_{\mathrm{e}}(p_{il}).$$

The above formulation is inspired by the Fisher’s score. The highest FiRE scores are thus assigned to rarest of the expression profiles present in a scRNA-seq data.

### Parameter value selection for FiRE

The process of hashing cells to buckets is repeated *L* times. For obvious reasons, a large value of *L* ensures rareness estimates with low variance.

For every estimator, the Sketching technique randomly sub-samples a fixed set of *M* features. While a very small choice of *M* requires a commensurately large number of estimators, a very large *M* might make the FiRE scores sensitive to noisy expression readings.

For all experiments, the hash table size, i.e. *H*, was set 1,017,881. It should be a prime number large enough to avoid unwanted collisions between dissimilar cells. In practice, *H* is chosen to be a prime number greater than 10 times of the number of items to be hashed.

On two independent datasets, we experimented with different values of *L* and *M*. We found *L* = 100 and *M* = 50 were a reasonably good choice to be considered as default values of *L* and *M*, respectively (Supplementary Figure 3).

### IQR thresholding criteria for rare selection identification

FiRE marks a cell as rare if its FiRE score is ≥q3 + 1.5 × IQR, where q3 and IQR denote the third quartile and the interquartile range (75th percentile−25th percentile), respectively, of the number of FiRE scores across all cells.

### F_1_ score computation for the simulation study

Both RaceID^15^ and GiniClust^16^ provide a binary prediction for rare cells. The contamination parameter in scikit-learn package implementation of LOF gives a threshold for the identification of outliers. In a two-class experiment (293T and Jurkat cells), it is straightforward to construct a confusion matrix. The F_1_ score on a confusion matrix can easily be computed as follows:

$${\mathrm{F}}_1{\mathrm{score}} = 2\frac{{{\mathrm{precision}} \times {\mathrm{recall}}}}{{{\mathrm{precision}} + {\mathrm{recall}}}}.$$

For the simulation experiment, rare cells were considered ones whose FiRE scores satisfied the IQR thresholding criterion.

For all algorithms, the F_1_ score has been calculated with respect to the minor population of the Jurkat cells.

### Identification of differential genes

A traditional Wilcoxon’s rank sum test was used to identify DE genes with an FDR cutoff of 0.05 and an inter-group absolute fold-change cutoff of 1.5. Fold-change values were measured between group-wise mean expression values of a given gene. We qualified a gene to be a cell type-specific one if it was found differentially up-regulated in a particular cluster, as compared to each of the remaining clusters.

### ISH data from Allen Mouse Brain Atlas

We cross-referenced a number of genes specific to the newly discovered cell types with ISH data of mouse brain cells from the Allen Mouse Brain Atlas^20^. One must note that such cross-referencing is of limited utility. Images depicting ISH-based expression measurements mark the enrichment and abundance of a certain gene in a specific anatomical area under study. It is usually impossible to zero down on the cell type.

### Identification of doublets

We used DoubletFinder^27^ to select top thousand putative doublets from the ~20 k mouse brain data^5^. The below command was used to identify the doublets:

doubletFinder(seu, expected.doublets = 1000, proportion.artificial = 0.25, proportion.NN = 0.01)

### Time complexity analysis

Performance measures reported in this article are recorded on a workstation with 40 cores using Intel Xeon E7-4800 (Haswell-EX/Brickland Platform) CPUs with a clock speed of 1.9 GHz, 1024 GB DDR4-1866/2133 ECC RAM and Ubuntu 14.04.5 LTS operating system with 4.4.0-38-generic kernel. The time taken by each algorithm has been measured by running it on a single core.

### Code availability

FiRE software package is available at: https://github.com/princethewinner/FiRE.

## Electronic supplementary material

Supplementary Information

## Data Availability

The study uses various publicly available scRNA-seq datasets. Both PBMC and 293T–Jurkat cell data that support the findings of this study are available from https://support.10xgenomics.com/single-cell-gene-expression/datasets. The preprocessed mouse brain data profiled from the Arc-ME region is available from https://portals.broadinstitute.org/single_cell. The mouse ESC dataset can be accessed at the GEO under accession code GSE65525. The mouse intestinal organoid cell data can be accessed at the GEO under accession code GSE62270.
