# Supplementary material for: Discovery of rare cells from voluminous single cell expression data
Source: Nat Commun. 2018 Nov 9;9:4719. doi: 10.1038/s41467-018-07234-6 (PMC6226447; doi:10.1038/s41467-018-07234-6)
Supplement: Supplementary file 1 — Supplementary Information [file 41467_2018_7234_MOESM1_ESM.pdf]

Discovery of rare cells from voluminous single cell expression data

Supplementary Material

Jindal et al.

Supplementary Methods

FiRE - Pseudocode

Algorithm 1 FiRE

```
function FiRE(Input: dataset) # cells x genes
    nSamples ← Number of data samples (cells)
    nFeatures ← Number of features (genes)
    X[nSamples][nFeatures] ← dataset
    L ← Number of runs
    M ← Number of dimensions to be sampled
    H ← Number of bins
    bins[L][H][.] ← Keeps the bin details across runs
    neighborhood[nSamples][L] ← Stores the size of the neighborhood of each sample
    scores[nSamples] ← Stores the score for each sample
    mi ← min(X)
    ma ← max(X)
    for i = 1 to L do
        _index[nSamples] ← [0] # Keeps the bin index for all the sample points for the current run.
        for j = 1 to M do
            _ind ← randomInt(low = 1, high = nFeatures, dist = "Uniform")
            _th ← randomFloat(low = mi, high = ma, dist = "Uniform")
            _pr ← randomInt(low = 1, high = maxInt, dist = "Uniform")
            for k = 1 to nSamples do
                _v ← 0
                if X[k][_ind] >= _th then
                    _v ← 1
                _index[k] ← _index[k] + _pr * _v
        for k = 1 to nSamples do
            _index[k] ← _index[k]%H
            bins[i][_index[k]].append(k)
    for i = 1 to L do
        for j = 1 to H do
            _b ← bins[i][j]
            _l ← length(_b)
            for k = 1 to _l do
                neighborhood[_b[k]][i] ← log(_l/nSamples)
    for k = 1 to nSamples do
        _t ← 0
        for i = 1 to L do
            _t ← _t + neighborhood[k][i]
        scores[k] ← −2 * _t
    return scores
```

Supplementary Figures

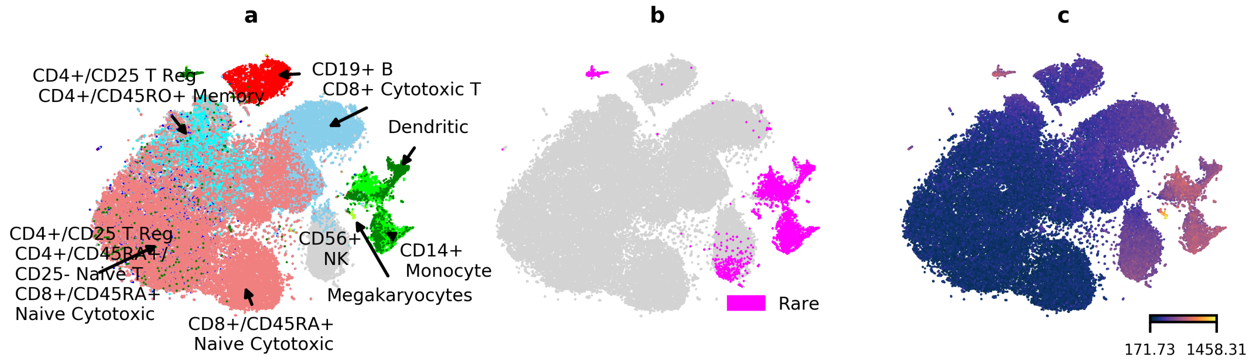

Supplementary Figure 1. Performance evaluation of FiRE on PBMCs. (a) t-SNE based 2D embedding of the data with color coded cluster identities as reported by Zheng and colleagues[1]. (b) Rare population identified by FiRE using IQR-thresholding-criteria. (c) Heat map of FiRE scores for the individual PBMCs. The cluster of megakaryocytes (0.3%), the rarest of all the cell types are assigned the highest FiRE scores.

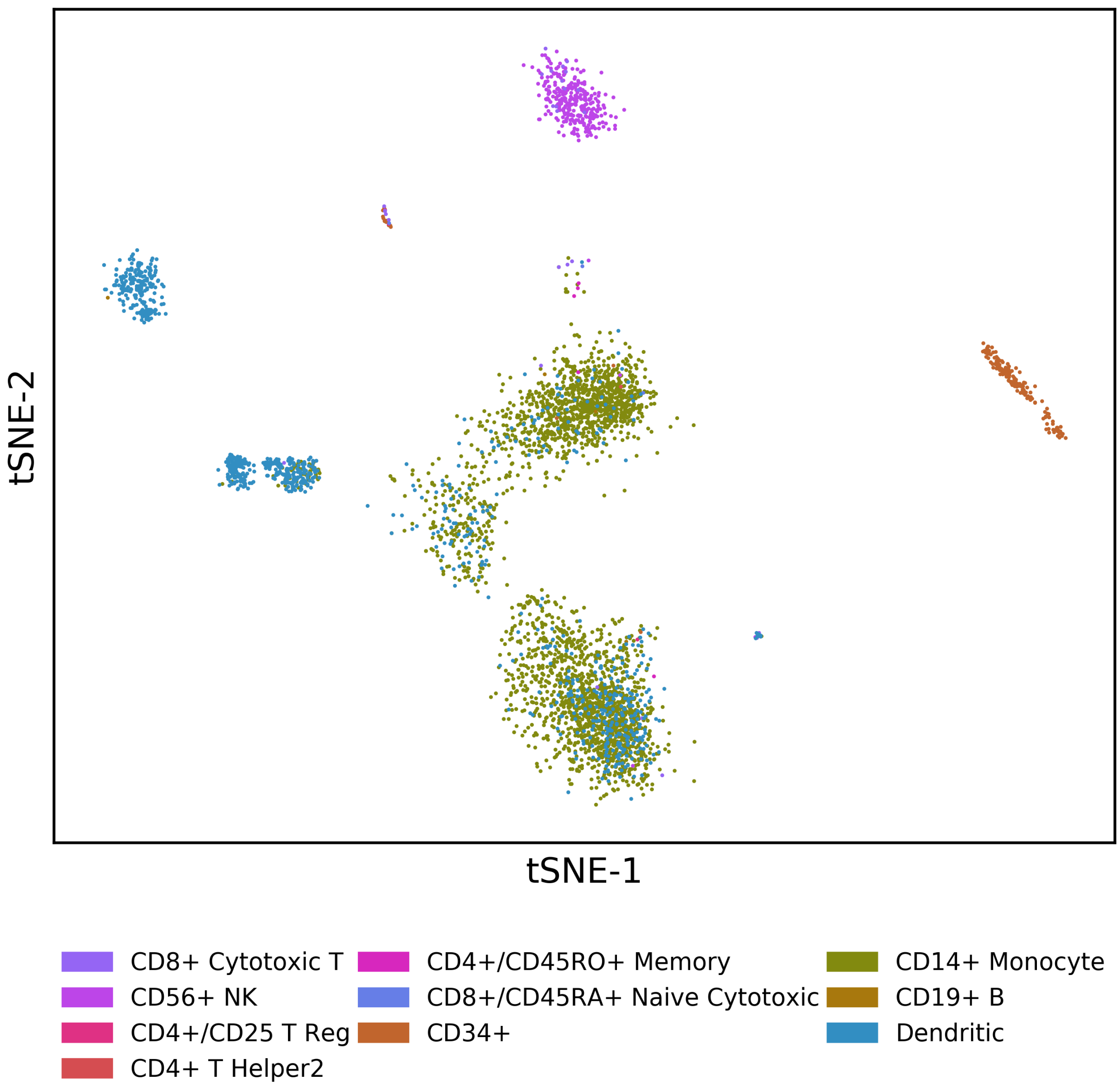

Supplementary Figure 2. 2D embedding of rare cells detected by FiRE on ∼68k PBMCs. Cells are color coded based on the cell type annotations reported by Zheng[1].

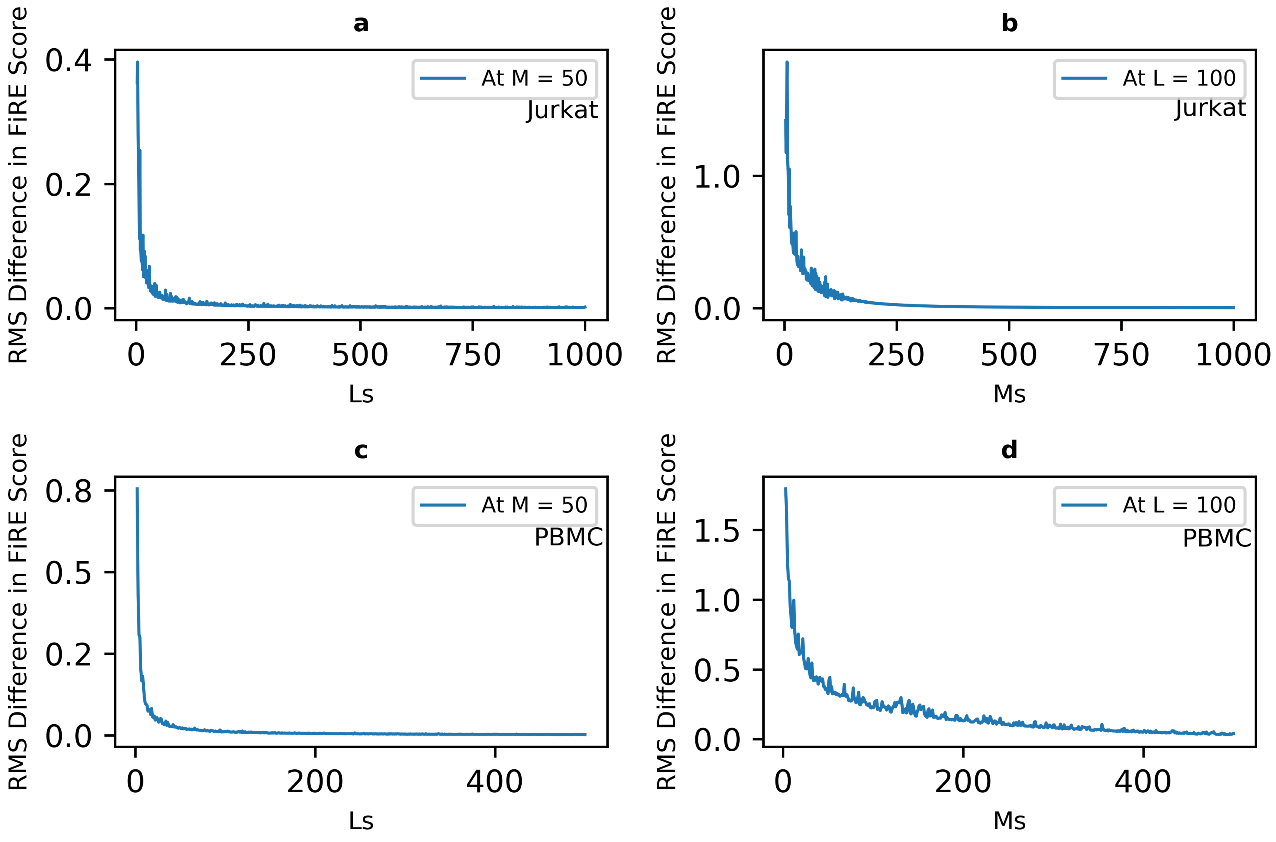

Supplementary Figure 3. Stability of FiRE. (a),(c) Root Mean Square (RMS) difference in values of FiRE score of every cell between two successive estimators. For calculation of RMS, FiRE score is normalized by the value of L. (b),(d) RMS difference in values of FiRE score between two successive values of M. For calculation of RMS, FiRE score is normalized by the value of M. (a)-(b) RMS has been shown on a simulated dataset consisting of a mixture of Jurkat and 293T cells[1]. (c)-(d) RMS has been shown on ∼68k Peripheral Blood Mononuclear Cells (PBMCs).[1]

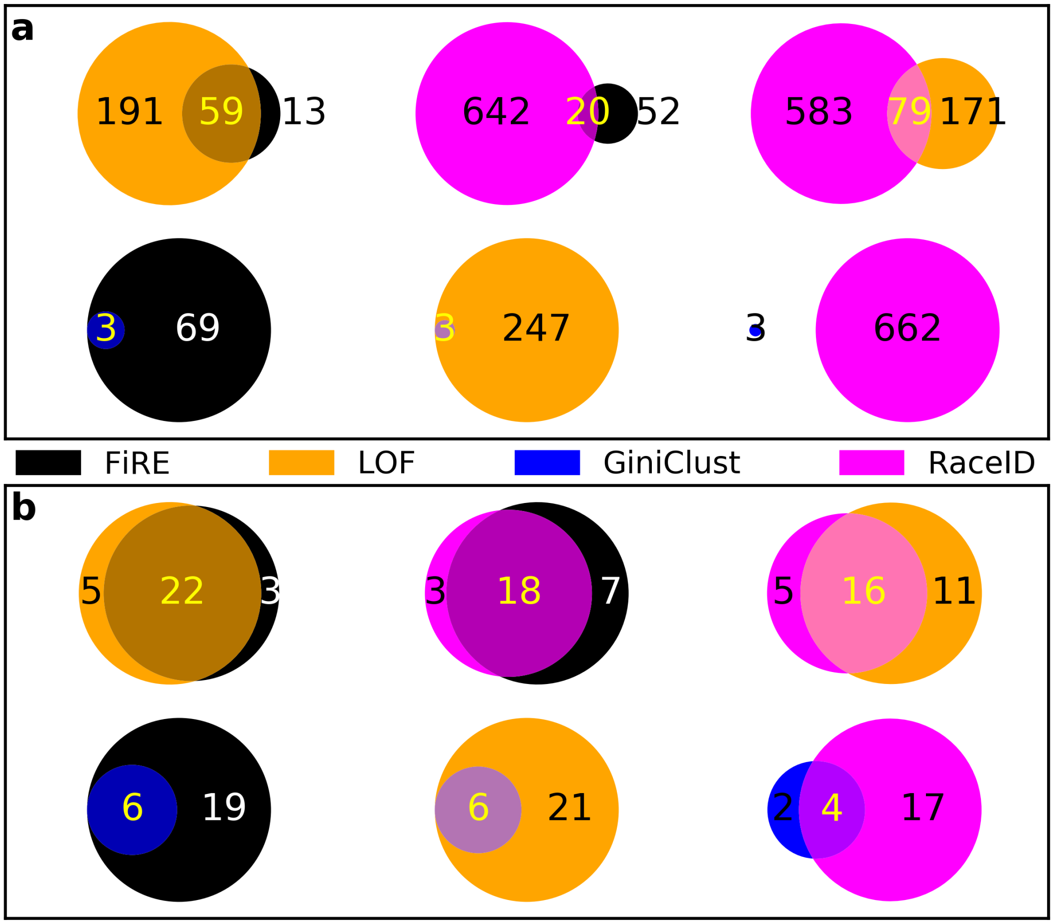

Supplementary Figure 4. Congruence of methods using Venn diagrams. (a),(b) Performance comparison of FiRE, GiniClust[2], RaceID[3] and LOF[4] as per the rare cells identified by them. (a) Performance comparison of methods above on Embryonic Stem Cells (ESCs) data[5]. FiRE could easily identify the *Zscan-4* enriched, 2C-like cell cluster as reported by Jiang *et al.*[2]. Also, the FiRE predicted rare cells had the least overlap with the ones predicted by RaceID, which could not identify those 2C-like cells. (b) Performance on mouse small intestine cells[3]. FiRE could identify the rare cell types in the secretory lineage, which consisted of goblet, enteroendocrine, paneth and tuft cells (as discussed in Grun *et al*[3]).

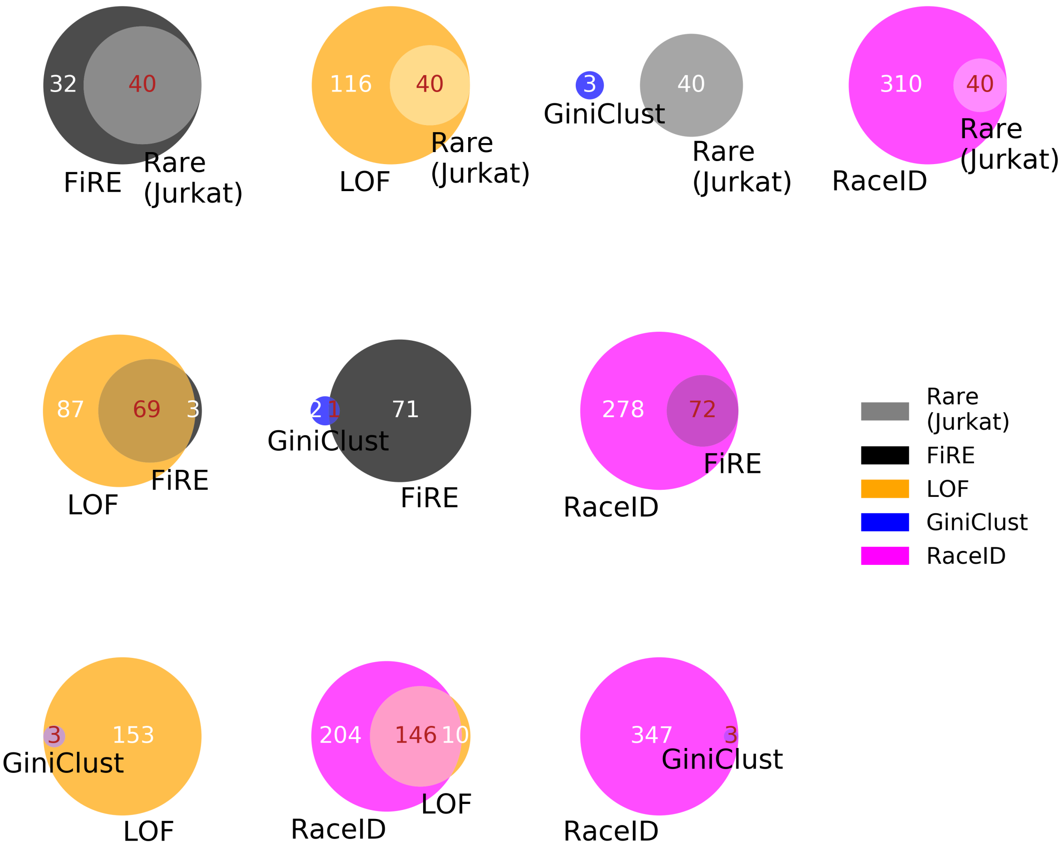

Supplementary Figure 5. Congruence of methods. Congruence of methods with known annotations and congruence between pairs of methods on a simulated, scRNA-seq data consisting of 293T and Jurkat cells mixed *in vitro* in equal proportion (Methods)[1].

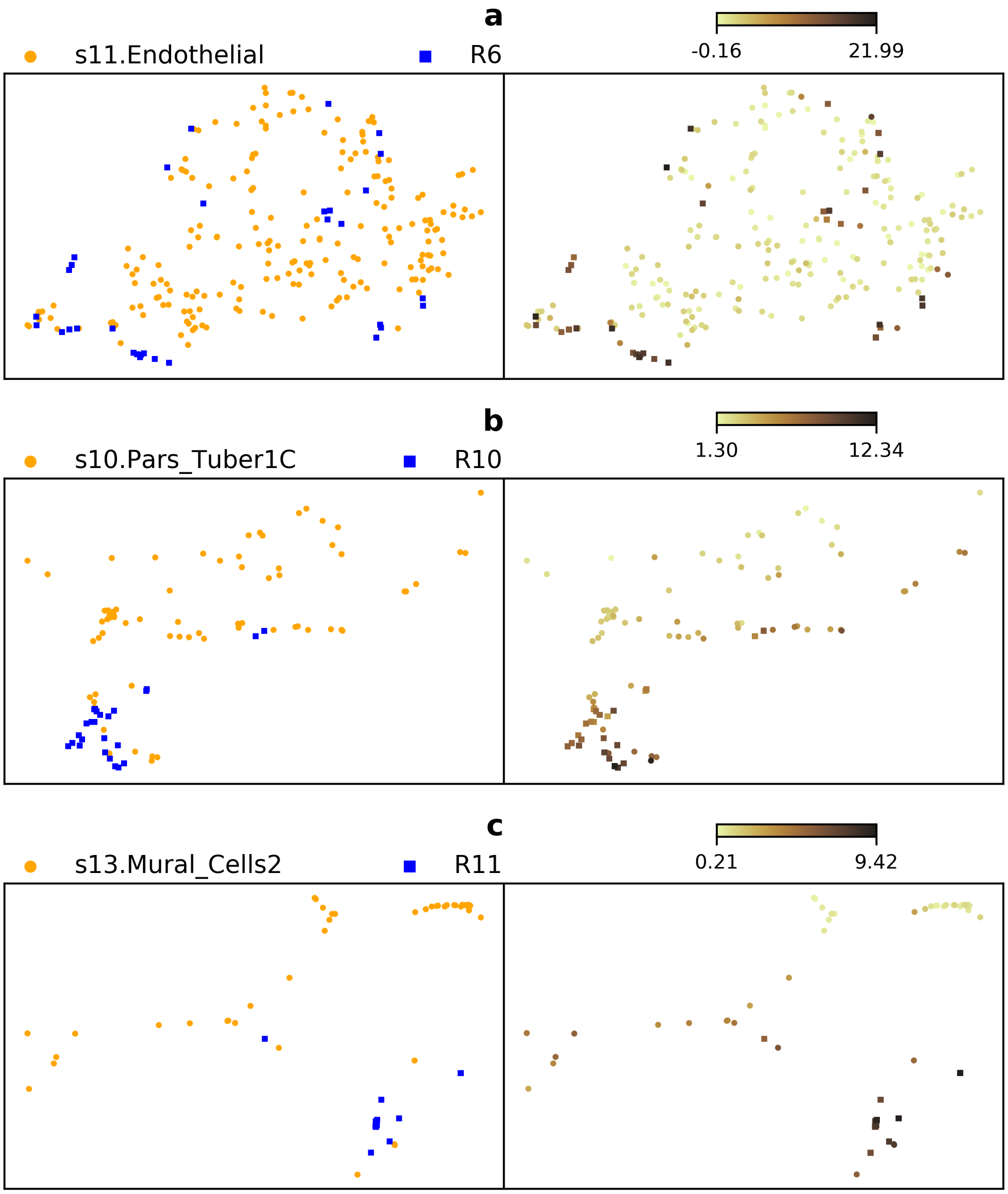

Supplementary Figure 6. Cell-subpopulations identified by FiRE. The left-hand side figure of each panel shows cells from one of the rare cell types and their parent cluster, coded by different colors. The right-hand side heatmap shows the average values of z-scores of differentially up-regulated genes in a predicted minor cell-subpopulation. R6, R10, and R11 appeared as sub-types of endothelial, pars tuberalis, and mural cells respectively, as reported by Campbell and colleagues[6].

Supplementary Tables

Samples	FiRE time (min)	LOF time (min)	GiniClust time (min)	RaceID time (min)
1000	0.0289066871	0.0442489147	0.0517063022	26.14682858
2500	0.0365521709	0.250727284	0.085117805	544.0205631
5000	0.0502097885	0.985178566	0.2058585723	4729.290497
10000	0.0783540368	3.79507608	0.6036896904	-
15000	0.1040728211	7.898171449	1.187051054	-
20000	0.1308689475	14.83272862	2.456504417	-
25000	0.1575588862	21.9108673	3.621491746	-
30000	0.1842079679	28.68896627	6.329408483	-
35000	0.2123669028	43.65191623	7.754190397	-
40000	0.2424690485	53.09929506	10.74303849	-
45000	0.2728209337	67.55955516	13.65257459	-
50000	0.2950131019	81.28970995	-	-
55000	0.3231681029	91.62621465	-	-
60000	0.3635717352	108.2481951	-	-
65000	0.3853084048	145.867392	-	-
68579	0.4325375557	160.8379446	-	-

Supplementary Table 1. FiRE is fast. Execution time (in minutes) recorded for the four methods while varying the number of cells from 1k to ~68k.

	Predicted Rare Cluster #	R1	R2	R3	R4	R5	R6	R7	R8	R9	R10	R11	R12
Second Level cluster	Total # Cells in Second Level Cluster												
s01.Ependymocy1	37	30											
s02.Ependymocy2	430	121		1									
s03.Oligodendro1	23				7								
s04.Oligodendro2	12				6								
s05.Oligodendro3	96				15								
s06.Oligodendro4	392				36								
s07.Oligodendro5	39				17								
s08.Pars_Tuber1A	42					31							
s09.Pars_Tuber1B	15					1					1		
s10.Pars_Tuber1C	93					15					24		
s11.Endothelial	240		144				32	1					
s12.Mural_Cells1	29							19				1	
s13.Mural_Cells2	55	1	5					4				11	
s14.PVMs	26									6			
s15.Microglia	304									15			
s16.Fibroblasts1	14												11
s17.Fibroblasts2	38												
s18.Fibroblasts3	120		1					1					
s19.Parstuber2A	230					2							
s20.Parstuber2B	8					1							
s21.Neurons1	37												
s22.Neurons2	502												
s23.Neurons3	693												
s24.Neurons4	533												
s25.Neurons5	799												
s26.Neurons6	10515					1				1			
s27.Oligodendro6	185			1	8								
s28.NG2_OPC1	23												
s29.NG2_OPC2	604			3						1			
s30.b2_Tanycytes1	592			4					14				
s31.b2_Tanycytes2	592			2					14				
s32.Astrocytes	99	2		11									
s33.a1_Tanycytes2	68			2									
s34.a1_Tanycytes1	334	12		21									
s35.b1_Tanycytes	1933			50									
s36.a2_Tanycytes	1169			20									
TotalCells/Percentage	20921	0.79346	0.71698	0.54969	0.42541	0.24377	0.15296	0.1195	0.13384	0.10994	0.1195	0.05736	0.05258

Supplementary Table 2. Distribution of cell-subpopulations identified by FiRE. Each row represents the subpopulations identified by Campbell and colleagues[6]. Columns, R1 to R12, represent the minor cell-subpopulations identified by FiRE, when clustered using dropClust[7].

Supplementary References

1. Zheng, G. X. Y. *et al.* Massively parallel digital transcriptional profiling of single cells. *Nat. Commun.* **8** (2017). DOI 10.1038/ncomms14049.
2. Jiang, L., Chen, H., Pinello, L. & Yuan, G. . Giniclust: Detecting rare cell types from single-cell gene expression data with gini index. *Genome biology* **17** (2016). DOI 10.1186/s13059-016-1010-4.
3. Grün, D. *et al.* Single-cell messenger rna sequencing reveals rare intestinal cell types. *Nat.* **525**, 251–255 (2015). DOI 10.1038/nature14966.
4. Breunig, M. M., Kriegel, H.-P., Ng, R. T. & Sander, J. Lof: Identifying density-based local outliers. *SIGMOD Rec.* **29**, 93–104 (2000). DOI 10.1145/342009.335388.
5. Klein, A. *et al.* Droplet barcoding for single-cell transcriptomics applied to embryonic stem cells. *Cell* **161**, 1187 – 1201 (2015). DOI 10.1016/j.cell.2015.04.044.
6. Campbell, J. N. *et al.* A molecular census of arcuate hypothalamus and median eminence cell types. *Nat. neuroscience* **20**, 484 (2017). DOI 10.1038/nn.4495.
7. Sinha, D., Kumar, A., Kumar, H., Bandyopadhyay, S. & Sengupta, D. dropclust: Efficient clustering of ultra-large scrna-seq data. *Nucleic Acids Res.* **46**, e36 (2018). DOI 10.1093/nar/gky007.
